# Potential pre-activation strategies for improving therapeutic efficacy of mesenchymal stem cells: current status and future prospects

**DOI:** 10.1186/s13287-022-02822-2

**Published:** 2022-04-04

**Authors:** Meirong Li, Yufeng Jiang, Qian Hou, Yali Zhao, Lingzhi Zhong, Xiaobing Fu

**Affiliations:** 1 Research Center for Tissue Repair and Regeneration Affiliated to the Medical Innovation Research Division and 4th Medical Center, PLA General Hospital and PLA Medical College, Beijing, China; 2 PLA Key Laboratory of Tissue Repair and Regenerative Medicine and Beijing Key Research Laboratory of Skin Injury, Repair and Regeneration, Beijing, China; 3 Research Unit of Trauma Care, Tissue Repair and Regeneration, Chinese Academy of Medical Sciences 2019RU051, Beijing, China; 4 Central Laboratory, Trauma Treatment Center, Chinese PLA General Hospital, Hainan Hospital, Sanya, China; 5 Wound Repairing Department, PLA Strategic Support Force Characteristic Medical Center, Beijing, 100101 China

**Keywords:** Mesenchymal stem cell, Pre-activation, Therapeutic potential, Individualized MSCs therapy

## Abstract

Mesenchymal stem cell (MSC)-based therapy has been considered as a promising approach targeting a variety of intractable diseases due to remarkable multiple effect of MSCs, such as multilineage differentiation, immunomodulatory property, and pro-regenerative capacity. However, poor engraftment, low survival rate of transplanted MSC, and impaired donor-MSC potency under host age/disease result in unsatisfactory therapeutic outcomes. Enhancement strategies, including genetic manipulation, pre-activation, and modification of culture method, have been investigated to generate highly functional MSC, and approaches for MSC pre-activation are highlighted. In this review, we summarized the current approaches of MSC pre-activation and further classified, analysed the scientific principles and main characteristics of these manipulations, and described the pros and cons of individual pre-activation strategies. We also discuss the specialized tactics to solve the challenges in this promising field so that it improves MSC therapeutic functions to serve patients better.

## Introduction

**Fig. 1:**
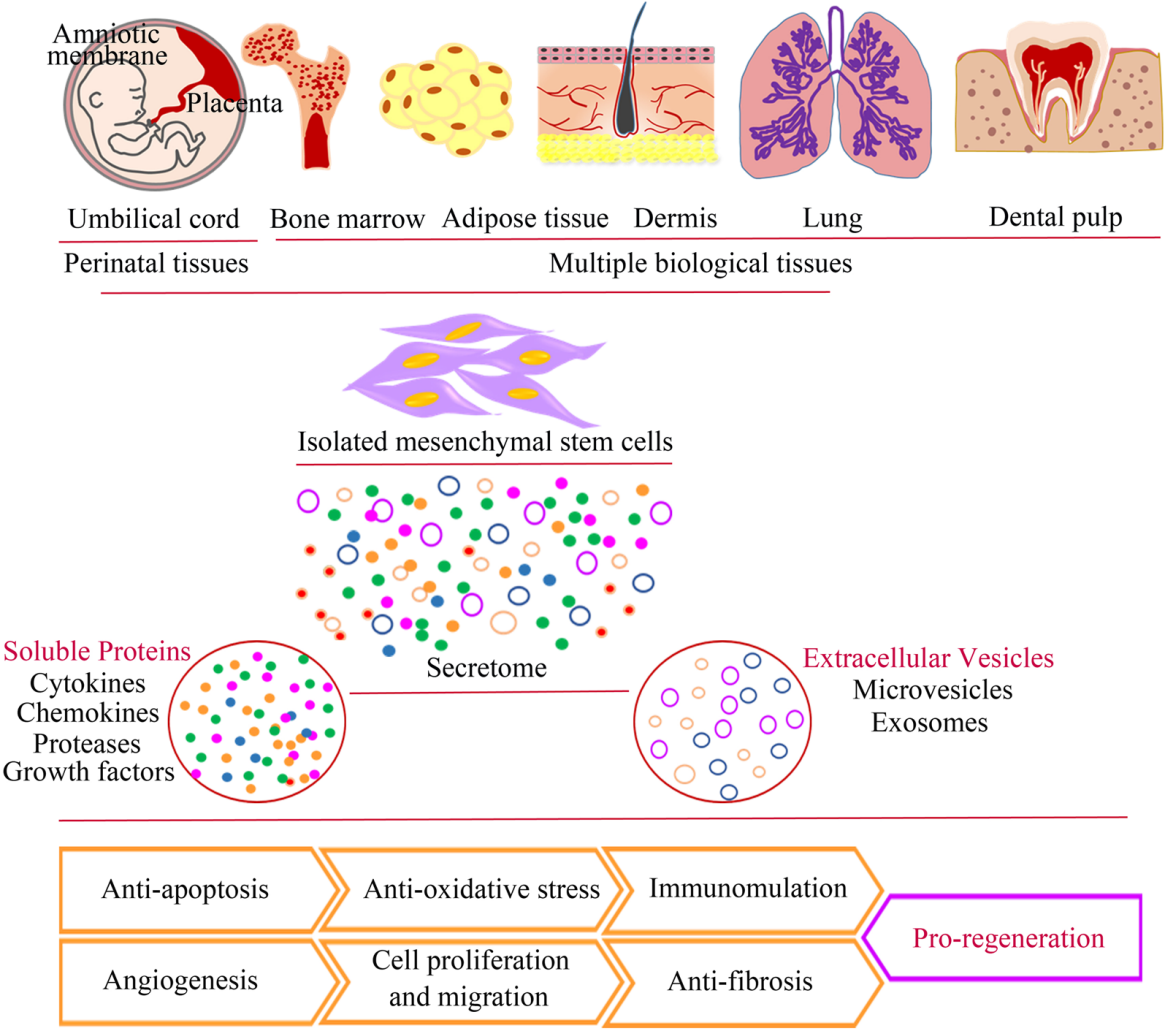
Isolation sources and action mechanisms of MSCs. The diagram illustrates various sources of isolated MSCs and the biological characteristics related to the therapeutic effect

In recent decades, MSCs in cell-based therapy have spanned across various diseases in experimental and clinical researches worldwide, exhibiting therapeutic efficacy over conventional treatments due to their distinctive biological properties [1–5]. They have isolated from perinatal tissues, such as umbilical cord, umbilical cord blood and placenta, and multiple biological tissues in adults, including bone marrow, adipose tissue, muscle, and lung [6, 7]. MSCs, as a kind of multipotent stromal cells, possess the potential for self-renewal and multilineage differentiation into adipocytes, muscles, chondrocytes, osteoblasts, and neuronal cells [8, 9]. In addition, increasing evidence has revealed that MSCs exert immunomodulation, reparative, and regenerative effects through high paracrine activity [10–12] (Fig. 1). More importantly, MSCs are immune privileged, which means allogeneic MSCs transplantation will not elicit inflammatory response, mainly due to their lack of class-II major histocompatibility complex (MHC-II) and costimulatory molecules [13, 14]. Their outstanding features jointly make MSCs the ideal seed cells in cell therapy after haematopoietic stem cells.

However, MSCs from different individuals are heterogeneous in their biological effects. Moreover, the body's internal environment (sick or not, youth or old) affects the quality of the isolated stem cells. For example, adipose-derived MSCs (ASCs) isolated from obese and type 2 diabetes (T2D) individuals exhibit functional defectives, such as increased apoptosis, reduced immunosuppressive activities, and loss of stemness [15–17]. And MSCs from old donors show impairment of proliferation and differentiation, depression of immunoregulation, and reduced secretion of bioactive molecules [18]. In addition, once administered in the body, MSCs may undergo apoptosis shortly because they exposed to the harsh host microenvironment, including hypoxia, oxidative stress as well as chronic inflammation. It reported that only about 28% of the intravenously injected MSCs survived after one day, [19] and fewer than 1% of cells persisted more than a week [20, 21]. Even if transplanted in situ, most MSCs lose their biological activities within one week [22]. Besides, most of the infused MSCs trapped in the lung microvasculature instead of the target tissues [23]. These adverse conditions will cause various problems, such as low survival rates of the transplanted cells, poor migration and homing of MSCs, and limit the functionalities of the injected cells.

To achieve the desired therapeutic potential, it seems unreasonable to increase the dosage and frequency of transplanted MSCs, as this may increase the risk of pulmonary embolism and the cost [24]. Optimizing the potency and therapeutic benefits of MSCs is a top priority. Several strategies attempted to optimize stem cells were proposed, which roughly divided into two categories, namely genetic modification and non-genetic modification (pre-activation).

In terms of genetic modification, MSCs will produce or overexpress functional genes that enable them to resist hostile microenvironment and apoptosis, increase migration and homing, and enhance paracrine effects. Several studies suggest that gene transfected MSCs have better therapeutic potential than wild-type MSCs [25–27]. However, safety is the greatest barrier for the future clinical therapeutic use of genetically modified MSCs. It reported that viral expression systems can elicit immune and inflammatory responses in the host, and viral integration in the host genome poses a tumourigenic risk [28, 29]. Additionally, the therapeutic potential and long-term function improvements in genetically engineered MSCs need to fully elucidate. Therefore, the development of highly efficient non-genetic modification methods, collectively referred to here as pre-activation, is an alternative and operational way to improve the treatment outcome of MSCs.

**Fig. 2:**
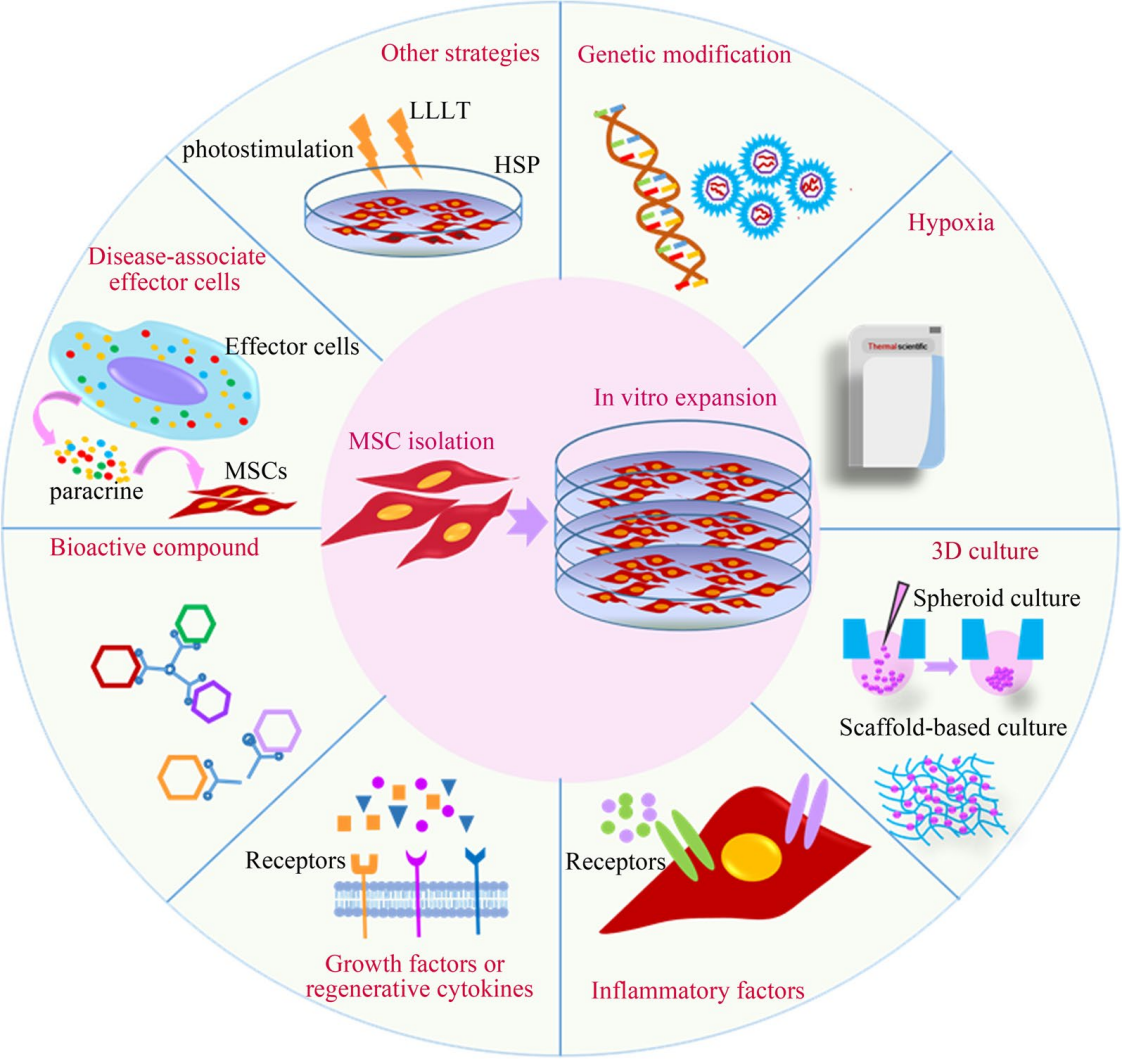
An overview of currently available strategies for preconditioning MSCs to improve the beneficial therapeutic effects of MSCs

MSCs can be pre-activated to achieve the desired function and reverse their inactivation because they can recognize the stimuli in the microenvironment and remember them [3, 30]. Reviewing the current literature, the pre-activation of MSCs is mainly based on the in vivo physiological microenvironment of MSCs survival and simulated in vitro, which is called "physiological microenvironment simulation pre-activation." Or in vitro adaptive regulation of MSCs is based on the pathological microenvironment of the disease, known as "pathological microenvironment simulation pre-activation." The primary goal of the review article is to provide specific methods involving in both types of pre-activation (Fig. 2).

## Methods

The most relevant English papers were selected by the distinct keywords, including mesenchymal stem cell and pretreatment, in the database of Google Scholar, MEDLINE, PubMed, and Embase. We set dates of searching from 2010 to 2021. An initial repeat assessment of selected articles was performed using endonote. Titles, abstracts, and full-text articles are further screened independently by two reviewers, and articles relevant to the topic of the current review were included. We also studied the references of the relevant papers, according to the needs of the writing.

## Physiological microenvironment simulation pre-activation

The number of MSCs in primary culture is limited. It needs to expand in vitro to achieve therapeutically relevant cell numbers, and excessive ex vivo manipulation leads to senescence, decreased stemness, and impaired regenerative capacity [23]. Maintaining the “youthfulness” of MSCs in vitro is very important. Stem cells live in specific areas of tissues, named as stem cell niche. It is a multidimensional environment composed of both cellular and acellular components to manipulate stem cell proliferation, determine stem cell fate, and maintain stem cell homeostasis. The cellular and acellular components involved in a number of necessary clues, namely other cells (cell–cell interactions), physical elements (temperature, osmotic pressure, stretch, and electrical signals), chemical factors (PH, oxygen, nutrients, ionic strength, metabolites cytokines, and chemokines), and extracellular matrix (composition, structure, topology, and stiffness) [31–33]. In this regard, recreating the complex in vivo microenvironment in vitro would provide a powerful tool for MSC production and maintain their inherent biological properties. And hypoxic and three-dimensional (3D) culture is by far the most intensively studied.

### Pre-activation with hypoxia

Under in vitro culture conditions, MSCs commonly exposed to an environment where the average oxygen tension is approximately 21% [34]. However, MSCs generally reside in a hypoxic microenvironment with physiological oxygen concentrations ranging from 1 to 11% in vivo [35, 36]. Several studies have illustrated that high oxygen concentration causes environmental stress in cultured MSCs, and then induces DNA damage and senescence [37, 38], and decreases their activities [39, 40]. Therefore, hypoxia is a crucial component of the physiological microenvironment for MSCs. Hypoxia-inducible factors (HIFs), especially HIF-1, are the crucial modulator of cellular response to hypoxia [41]. HIF-1 is a heterodimer containing two subunits, HIF-1α and HIF-1β. The lack of O_2_ allows oxygen labile protein HIF-1α accumulation and translocates into the nucleus, and then binds with HIF-1β to form the heterodimer, which further binds to a hypoxia-response element (HRE) in the target genes with co-activators such as CBP/p300 and then regulates the transcription of numerous genes [42–44].

Hypoxic pre-activation has multiple beneficial effects on MSCs. For example, a hypoxic culture environment maintains undifferentiated states of MSCs. Several reports showed that low oxygen tension increased the expression of multipotent stem cell markers (Oct4, Sox2, and Nanog) in ASCs without changing their surface markers and morphology [45, 46].

In addition, hypoxia facilitates the proliferation and survival of MSCs, leading to a higher expansion and more yield ASCs compared to normoxic state (20% O2) [45–48]. And the hypoxia priming seems to promote the mobilization of MSCs in vitro in migration assay [47, 49] and improve homing of MSCs in vivo [50, 51]. Lee et al. group demonstrated that hypoxia promoted the proliferation and migration potential of MSCs through the HIF-1α-GRP78-Akt signal axis [34]. The results of Rosová et al. showed that MSC cultured in hypoxia augmented the expression of hepatocyte growth factor (HGF) and its major receptor cMet, and HGF/cMet was the main signalling for MSC migration [51]. Another study found that hypoxic pre-activation promoted the migration of bone marrow-derived mesenchymal stem cells (BMSCs) by ways of increasing potassium Kv2.1 channel expression and FAK activities [50].

Besides, hypoxia priming could protect MSCs against the hostile microenvironment and maintain gene stability [37, 52]. The accumulation of HIF-1α under hypoxia activates normal cellular prion protein, which protects MSCs from oxidative stress-induced apoptosis via the activation of superoxide dismutase and catalase to inactivate cleaved caspase-3 [53]. Furthermore, when exposed to hypoxia, MSCs show a prolonged life span and avoid replicative senescence, and express fewer senescence-associated β-galactosidase compared to normoxic culture [53]. Hypoxia-conditioned MSCs exhibit gene stability with decreased DNA damage and less chromosomal aberration [37].

Moreover, the secretion profile of MSCs changes and the paracrine function is enhanced, after hypoxic culture. For instance, under hypoxia, the secretion of pro-angiogenic factors such as VEGF, HGF, and fibroblast growth factor-basic (bFGF) increased in MSCs. In contrast, anti-angiogenic factors such as thrombospondin-1 and plasminogen activator inhibitor-1 decreased in MSCs [3]. Furthermore, the immunosuppressive characteristics of MSCs improved under hypoxia with upregulation of anti-inflammatory factors including interleukin-6 (IL-6), IL-10, and indoleamine 2,3-dioxygenase (IDO) [54, 55]. And, hypoxia-cultivated MSCs inhibited the proliferation of CD4 and CD8 T lymphocytes and promoted the generation of Treg cells more effectively than MSCs exposed to normal oxygen [56, 57].

Significantly, numerous experimental studies found that MSCs pre-activated with hypoxia shows more prominent therapeutic effects than untreated MSCs. Hypoxia-pre-activated MSCs could obviously attenuate pulmonary oedema, alleviate pulmonary fibrosis, and improve lung function, compared to normoxic cultured MSCs in a pulmonary fibrosis model [58]. In addition, transplantation of hypoxic MSCs into a rat myocardial infarction model led to greater vascularization and smaller infarct size in injured sites compared to normoxic MSCs [59]. Furthermore, streptozotocin-induced diabetic mice received hypoxia-pre-activated MSCs caused significantly lower random and fasting blood glucose as well as improved oral glucose tolerance compared to normoxic MSCs-treated diabetic mice [40].

Therefore, compared with normoxic MSCs, MSCs pretreated with hypoxia present more favourable properties and hold better therapeutic potential. Additionally, hypoxic pre-activation has the advantages of being simple, low cost, easy popularization, and suitable for large-scale cell production. However, several issues need to be addressed before further application of hypoxic MSCs into the clinic. It is necessary to optimize the oxygen concentration in hypoxic pre-activation because the oxygen concentrations in physiological niches where MSCs live vary by tissue origin (1% to 7% in bone marrow, 10% to 15% in adipose tissue, and 1.5% to 5% in birth-associated tissues) is [58]. Moreover, the optimal duration for hypoxia also should be revealed. More importantly, biosafety is the most concerning issue of stem cell-based therapy and should be carefully determined before clinical application.

### Pre-activation with 3D culture

Compared with the two-dimensional (2D) culture environment, the 3D culture system imitates the natural MSC microenvironment in vivo and provides enhanced cell–cell interactions or cell-ECM interaction, which can significantly improve the biological behaviours of MSCs, such as proliferation, immune regulation, and committed differentiation [60]. The 3D culture systems of MSCs, including multicellular spheroids, scaffolds, and hydrogels, have attracted more and more attention.

#### Spheroid culture

Spheroid culture, a comparably easy method for strengthening the biological activities of MSCs, has established [61]. Various methods have developed for the generation of MSC spheroid, such as the hanging drop technique, low-attachment approach as well as forced aggregation techniques [62]. With the development of biomaterials, scaffold-based culture platforms to generate MSC spheroid have developed.

The spheroid culture system can benefit the therapeutic potential of MSCs through increasing stemness and facilitating differentiation into different cell lineages [57, 63]. The results of Cheng et al. showed that chitosan film-based spheroid culture could dedifferentiate MSCs into more primitive state with downregulation of mesenchymal lineage markers CD29, CD90, and CD105 and upregulation of pluripotency-related markers Sox2, Oct4, Nanog, and SSEA-4 [45, 64]. Moreover, Zhang et al. employed a microgravity bioreactor to generate MSCs spheroids and found similar results [65]. Furthermore, spheroid-derived MSCs alter their differentiation preference and can transdifferentiate into non-mesenchymal lineage cells such as neural cells and hepatocytes [64, 65].

The spheroid culture system can benefit the therapeutic potential of MSCs by enhancing proliferation, migration, and homing efficiency. Compared to monolayer MSC culture, spheroid-derived MSCs exhibited higher proliferative activity [66]. The SDF-1/CXCR4 signalling pathway plays crucial role in the migration and engraftment of transplanted MSCs. Culturing of MSCs as spheroid restores the loss of CXCR4 expression caused by 2D culture [67]. Beside, increased expression of matrix metalloproteinases MMP-9 and MMP-13 has found in spheroid-derived MSCs, facilitating cellular invasion via the basement membrane [66].

The spheroid culture system can benefit the therapeutic potential of MSCs through promoting the secretion of therapeutic factors, including immunomodulatory and pro-angiogenic cytokines. Bartosh et al. reported that MSCs in a hanging drop model secreted higher levels of the anti-inflammatory factors such as tumour necrosis factor-inducible gene 6 protein (TSG-6) and stanniocalcin 1, and more effective suppressed lipopolysaccharides (LPS)-stimulated macrophage secretion of tumour necrosis factor-alpha (TNF-α) than control MSCs [68]. Several studies have demonstrated that other anti-inflammatory cytokines such as prostaglandin E2 (PGE-2), transforming growth factor-β1 (TGF-β1) as well as IL-6 also exhibited a greater levels in spheroid derived MSCs [69–72] than 2D cultured MSCs. Furthermore, the pro-angiogenic and pro-regenerative function enhanced in spheroid derived MSCs due to the significantly increased secretion of VEGF, HGF, bFGF, and angiogenin (ANG) [67, 73].

Spheroid culture optimized the biological properties of MSCs and enables them better therapeutic effects in vivo. Bartosh et al. proved that spheroid-derived MSCs were more effective than monolayer MSCs in suppressing inflammatory responses in a zymosan-induced peritonitis model of mouse. Spheroid-derived MSCs treated group showed substantially decreased neutrophil activity and proinflammatory molecules in serum [70]. Cheng et al. reported that spheroid-derived MSCs injected into the impaired healing wounds significantly promoted healing rates by increasing cellular engraftment and enhancing angiogenesis [66]. The study of Bhang et al. demonstrated that compared with the control group, more significant angiogenesis and less fibrosis were observed in the ischemic region in the spheroid-derived MSC transplantation group [74].

However, some disadvantages of the spheroid culture system limit the large-scale production of MSC spheroids for in vivo applications [75]. For example, the spheroid culture technique affects the spheroid size, and the variability of spheroid size impacts the therapeutic performance of MSCs. Therefore, it is necessary to discriminate the clinical needs, develop a reproducible spheroid culture system, and utilize animal models and clinical trials to confirm its safety and effectiveness before using spheroid-derived MSC in clinical practice.

#### Scaffold-based culture

Extracellular matrix (ECM) is one of the critical acellular components of stem cell niche, which can considered as the “soil” for stem cells. Cells in the niche are mechanically anchored to the ECM through transmembrane proteins known as integrins [76]. The primary function of ECM is to provide the necessary scaffold for cell growth and transduction of mechanical sensing signals to cells through cell-ECM interaction. And it also supplies essential nutrients and growth factors to cells [61, 77]. Therefore, it is necessary to build engineered niches to simulate native ECM *in viv*o. Recent advances in biomaterials have contributed to the development of artificial ECM culture systems for MSCs, and a variety of natural and synthetic biomaterials have emerged [61]. They should fulfil the properties of matrix mechanics, degradability, and biocompatibility. And the design principles of engineered ECM mainly depend on the native microenvironment of the stem cells type of interest or the desired phenotypic output. The existing engineered ECM could improve the biological properties of MSCs such as proliferation, homing, lineage differentiation, and paracrine [78–81].

Engineered ECM in the forms of scaffold, film, hydrogel, and sponge has been developed and used to amplify MSCs and enhance their biological properties [82–84]. Alginate has extensively used as a biocompatible carrier in tissue engineering. The work of Ewa-Choy et al. documented that the 3D alginate hydrogels created a microenvironment that facilitated the differentiation of ASCs into chondrogenic-like cells in ASCs and nasal chondrocytes co-cultured system. The efficiency of ASC-chondrocyte differentiation depended on alginate concentration [85]. Of note, the addition of specific inducers to the ECM scaffold will further increase the differentiation efficiency of MSCs into desired cell types. Several studies have shown that hydroxyapatite (HAp) is similar to pre-existing minerals during the bone remodelling process and possessing superior osteoinductive activity [86, 87]. BMSCs were implanted into the porous sponge constructed from duck’s feet-derived collagen (DC)/ with or without hydroxyapatite (HAp) to observe their osteogenic differentiation. Under the induction of an osteoconductive regulator dexamethasone, BMSCs in DC/HAp sponge tended to show higher proliferative activity and greater osteogenic differentiation [88].

The paracrine profile and immunomodulatory roles of MSCs seeded on engineered ECM scaffolds are also significantly altered. MSCs seeded in biomaterials showed up-regulation of anti-inflammatory regulators such as PGE2 and TSG-6, and down-regulation of anti-inflammatory regulators such as monocyte chemoattractant protein-1 (MCP-1), IL-6, and receptor activator of nuclear factor κ-B (NF-κB) ligand (RANKL) [60, 89]. Alginate-encapsulated MSCs attenuate TNF-α secretion and enhance PGE2 production more effectively than MSCs in 2D in an LPS-stimulated model of organotypic hippocampal slice culture [90]. In addition, ASCs cultured in alginate hydrogels showed significant inhibition on the proliferation of phytohaemagglutinin-stimulated peripheral blood mononuclear cells compared to monolayer culture [91]. Moreover, alginate hydrogel encapsulated MSCs promoted the conversion of macrophages to the anti-inflammatory M2 phenotype in vitro. And they played a similar immunomodulatory role in a rat model of spinal cord injury (SCI) as a greater percentage of the M2 subsets at the site of injury compared to control [92, 93].

Therefore, scaffold-based 3D culture endowed MSCs with more excellent biological activities and outstanding therapeutic efficacy than 2D culture. With people's understanding of biomaterials, their biological performance is also constantly expanding. Using only biological materials, biomimetic composite materials with multiple functions can be manufactured. For example, Jiao et al. developed a double-phase biomimetic procallus with gelatin-reduced graphene oxide (GOG) and photo-crosslinked gelatin hydrogel, which provide hypoxic microenvironment and mediated bidirectional differentiation of BMSCs to osteogenesis and angiogenesis, thereby promoting the regeneration of bone defects [94]. Therefore, a new generation of scaffold-based MSC culture techniques should not only simulate stem cell niches from multiple aspects simultaneously, provide as many stem cell niche components as possible, but also release bioactive molecules to target effector cells and activate "self-repair" mechanisms at damaged sites.

## Pathological microenvironment simulation pre-activation

Since MSC enters the body, it will face the pathological microenvironment caused by the disease. There are a variety of destructive factors in this setting that can cause oxidative stress and apoptosis of transplanted cells, and significantly compromise the inherent therapeutic properties of MSCs. Researchers are committed to fine-tune the characteristics of the cells against hostile environments and suited for the targeted diseases. Based on the plasticity and memory ability of MSCs, cues in the trauma microenvironment, such as environmental factors (hypoxia), chemical factors (inflammatory factors and cytokines), will be the primary consideration for in vitro manipulation of MSCs [95]. In addition, new approaches have constantly explored, such as drugs, Traditional Chinese medicine and small molecule compounds have also become emerged for MSC priming [28, 96, 97]. Another concern is that the biological characteristics of endogenous MSCs in patients will change with the disease development, showing the loss and decline their function [98–100]. Therefore, it is meaningful to improve the biological activities of MSCs and enhance their therapeutic efficacy, especially to develop patient-customized therapeutic MSCs.

### Pre-activation of MSCs with hypoxia

It has mentioned in the previous sections that hypoxia is an important component of the stem cell niches. Actually, MSCs always delivered into the injury site of ischaemia and hypoxia in animal experiments and clinical studies. Therefore, hypoxia is also a crucial component of the pathological setting [34]. And hypoxic culture in vitro can induce the memory of MSC to injury microenvironment for better therapeutic efficiency, which have been discussed in previous sections. However, there is a difference in oxygen concentrations between stem cell niches and damaged tissue. Furthermore, local oxygen concentrations in damaged tissues vary during different repair periods. Thus, the oxygen concentration of stem cell pretreatment was varied by purpose. For example, the in vitro culture of MSCs refers to physiological oxygen concentrations and further pretreated with pathologic oxygen concentrations prior to their in vivo application.

### Pre-activation of MSCs with inflammatory factors

The pre-activation with inflammatory factors and cytokines is considered the most common means to mimic the inflammatory microenvironment in vivo and play a significant role in regulating the immunomodulatory function of stem cells [3]. In contrast with others, the proinflammatory cytokines such as TNF-α, INF-γ, and IL-1β are frequently observed in the traumatic microenvironment and are extensively studied for preactivating MSCs [3].

#### Pre-activation of MSCs with TNF-α

Increasing evidence suggests that appropriate priming of MSCs with disease-related stimuli improves their biological function and plays better therapeutic roles [101]. TNF-α is expressed in ischemic and injured tissues and commonly used to mimic the acute inflammatory environment [102]. Pre-activation of gingival tissue-derived MSCs (GMSCs) with TNF-α enhanced exosomal CD73 expression, which was essential for inducing anti-inflammatory M2 macrophage polarization [103]. Exosomes derived from TNF-α preactivated GMSC exhibited stronger anti-osteoclastogenic activity than control, thereby reducing periodontal bone resorption in a mice model of ligature-induced periodontitis [103]. Furthermore, TNF‐α- pre-activated MSCs showed improved proliferation, migration, and survival under H_2_O_2_‐-induced oxidative stress. And they exerted better endothelial protective functions through the massive secretion of HGF, VEGF and other cytokines than control MSCs [104]. Additionally, MSCs primed with TNF-α accelerated local vascularization of the injured sites in the ischemic hindlimb and cutaneous wound via secretion of pro-angiogenic cytokines, such as IL-6 and IL-8 [105, 106].

#### Pre-activation of MSCs with interferon (IFN)-γ

The proinflammatory cytokine IFN-γ is also a representative factor used for MSC pre-activation [107]. In response to IFN-γ, MSCs had a distinctive immunosuppressive profile, with the increased expression of several anti-inflammatory factors such as HGF, TGF-β1, IDO, prostaglandins, and cyclooxygenase 2 (COX-2) [108–110]. Prostaglandins and IDO secreted from the IFN-γ-stimulated MSCs were the main effectors in suppressing NK activation [109]. In addition, IL-2/15-activated NK cells induced less cytotoxicity to IFN-γ stimulated MSCs than nonstimulated MSCs due to their upregulation of inhibitory MHC Class I molecules, while IFN-γ-priming MSCs inhibited the proliferation of PBMCs more strongly than did the nonpriming MSCs [111], accompanied by upregulation of PD-L1 and increased secretion of COX-2-derived PGE2 [112]. The therapeutic potential of MSCs after IFN-γ pre-activation was significantly improved and demonstrated in models of CCl4-induced liver cirrhosis [113], obliterative bronchiolitis [114], and renal fibrosis [115]. This evidence illustrated that MSCs could be activated by inflammatory signalling, and sufficient to strengthen their immunoregulatory profile and therapeutic efficacy [110].

#### Pre-activation of MSCs with IL-1β

IL-1β is also a prevalent inflammatory cytokine in inflamed tissues produced by monocytes and macrophages [116]. It has shown that IL-1β pre-activation increases the expression of many adhesion molecules in MSCs, such as integrin LFA-1, thereby promoting adhesion to HUVECs through interaction with ICAM-1, which facilitates MSC cross-endothelium and homing [117]. In addition, Nie et al.’s study found that IL-1β pre-activated MSCs showed elevated CXCR4 expression and increased their migration towards SDF-1, leading to better therapeutic performance than naive MSCs in acute liver failure [118]. In addition, exosomes derived from IL-1β-pre-activated MSCs could induce macrophage polarization into M2 phenotype and attenuated the symptoms in the septic mice model more effectively than exosomes produced by naïve MSCs [119].

#### Pre-activation of MSCs with other proinflammatory cytokines and TLR ligands

In addition, some other inflammatory factors such as IL-17A and IL-25 were recently reported as alternative pre-activated means and acquired promising results. IL-17A is produced predominantly by CD4^+^ T helper 17 cells and plays regulatory roles in developing autoimmune and inflammatory diseases [120]. The results of a comparative study demonstrated that MSCs stimulated with IL-17A exhibited superior immunosuppressive properties than untreated MSCs [121]. IL-17A-treated MSCs showed the highest suppression of mitogen-activated CD3^+^ T cells compared with MSCs treated by IFN-γ, TNF-α, or IL-1β. And they also induce the generation of CD4^+^CD25^high^CD127^low^FoxP3^+^ Tregs [121]. IL-25 is a member of the cytokine IL-17 family and has recently used to enhance MSC regulated immune response [122, 123]. Infusion of IL-25-primed MSCs significantly reduced IL-17-positive cells and increased FoxP3 positive cells, thereby alleviating intestinal inflammation in a rat model of DSS-induced colitis compared with unprimed MSC [122].

Lipopolysaccharide (LPS) is a component of the outer membrane of gram-negative bacteria that elevated in various diseases. Recent studies have shown that LPS serves as an essential mediator in the regulation of apoptosis in numerous cell types [124]. It has demonstrated that the biological effects of LPS on MSCs were closely associated with the concentration LPS used. MSCs treated with LPS at high-dose induced their apoptosis, and MSCs treated with low-dose of LPS enhanced their ability to resist oxidative stress and inhibit apoptosis, possibly depending on the upregulation of cellular FADD-like IL-1β-converting enzyme inhibitory protein. Furthermore, transplantation of low-dose LPS preactivated MSCs significantly improved MSC-mediated cardio-protection in an I/R injury model through MyD88-dependent activation of stat3 [124–126].

#### Pre-activation of MSCs with a combination of proinflammatory cytokines

The biological activities of MSCs are varied after pre-activated with various proinflammatory factors, so scientists conceived whether MSCs can be pretreatment by the combination of different inflammatory cytokines to compensate for the lower efficiency and maximize the therapeutic effect. After licensing with IFN-γ and TNF-α, MSCs retained their anti-apoptotic ability, which inhibited T cell proliferation and promoted CD14^+^ monocytes differentiated into anti-inflammatory CD206^+^ M2 macrophages more effectively than single-factor-induced MSCs. Moreover, pre-activation of ASCs with a combination of IFN-γ, TNF-α, and IL-17 dramatically enhanced their immunosuppressive effect and effectively cured concanavalin A (ConA)-induced liver injury in mice through an iNOS-dependent manner [127].

Therefore, inflammatory cytokines pre-activation can not only improve the ability of MSCs to resist oxidative stress, but also largely enhance the immunosuppressive properties of MSCs and strengthen their therapeutic efficacy. Nevertheless, some questions still need to be answered. Intensive studies need to further explore and identify the optimal concentration and action duration of inflammatory cytokine pre-activation alone or in combination. Alternatively, the possible side effects of inflammatory pre-activation, such as undesirable upregulation of class I and II HLA molecules, should also be concerned.

### Pre-activation of MSCs with growth factors or regenerative cytokines

Priming MSCs with growth factors or regenerative cytokines have recently emerged and have proved to be an appealing approach. bFGF is one of potent pleiotropic cytokines and serves critical roles in regulating bio-properties of various stem cells and tissue regeneration [128]. bFGF-primed dental pulp stem cells derived from deciduous teeth (DPMSCs) show the highest angiogenic potential with the highest secretion of HGF and VEGF compared to control or hypoxic pre-activated MSCs [129]. Moreover, stimulation of canine MSCs with bFGF enabled their generation of cartilage tissue [130]. SDF-1 is also known as chemokine ligand 12 (CXCL-12), and its receptors CXCR4 and CXCR7 constitute the chemokine signalling critical for recruiting stem cells and organ repair after injury [131]. MSCs pre-activated with SDF-1 exhibited a significant anti-apoptotic capacity and proliferative potential, induced by a marked activation of Akt and ERK signalling pathways [132]. And angiogenesis also enhanced in preacitvated MSCs and was partly associated with their increased VEGF [132]. SDF-1α, the main spliced isoforms of SDF-1, has been used to priming MSCs [133]. SDF-1α pre-activation could augment the survival of MSCs in the infarcted myocardium, lessen the scar size, and enhance the cardiac systolic function [134]. In addition, infusion of MSCs pre-activated with TGF-α also obtained similar therapeutic effects in a rat model of acute myocardial I/R injury due to elevated VEGF secretion via a p38 mitogen-activated protein kinase (MAPK)-dependent mechanism [135].

Preactivating MSCs with a cocktail of growth factors revealed synergistic effects to enhance their biological function. Simultaneous pre-activation of MSCs with bFGF, IGF-1, and BMP-2 enhanced their plasticity and significantly upregulated myocardial transcription factors in the myocardial cells and MSCs co-culture model. Moreover, transplantation of these pre-activated MSCs resulted in reduced infarct size and improved cardiac function compared to transplantation of untreated MSCs [136]. However, selecting optimal cytokines to pre-activated MSCs and confer the desired biological function is a major step. Whether to choose cytokines that change in common in various diseases or to use disease-specific cytokines as a pre-activated condition is a question that needs to be thoroughly studied. Alternatively, the method of cytokine pre-activation is not economical enough because significant amounts of cytokines required for the large-scale production of pre-activated MSCs.

### Pre-activation of MSCs with bioactive compounds

Bioactive compounds are a promising pre-activation method for strengthening biological properties of MSCs [28, 96, 137]. At present, the bioactive compounds used stimulation of MSCs can divided into natural (such as extracted from Traditional Chinese medicine (TCM)) and synthetic compounds according to their original; in terms of the screening principle of bioactive compounds, they either have a biological regulation effect on MSCs or have a therapeutic effect on target diseases; given their biological mechanisms for MSCs, bioactive compounds can classified as follows: promotion of the survival and migration, enhancement of the secretory activity, and reversion and reparation of disabled MSCs.

#### Bioactive compound for promoting the survival and migration of MSCs

Practically, exerting the inherent therapeutic properties of MSCs requires the transplanted cells to survive and function in a harsh and damaged setting [28]. A number of studies have focused on modifying MSCs to enhance their anti-apoptosis and migration capacity by using bioactive compounds. Trimetazidine (1-[2,3,4-trimethoxybenzyl]piperazine; TMZ) can lower the tissue damage caused by ischaemia and usually used to treat angina. It protected MSCs from hydrogen peroxide (H_2_O_2_)-induced oxidative stress by increasing the expression of pro-survival factors such as HIF-1α, Akt, survivin, and Bcl-2. And a significant improvement in cardiac function was observed after transplantation of TMZ pre-primed MSCs in a myocardial infarction model [97]. Tadalafil belongs to the long-acting PDE5 inhibitor group and has been applied to treat heart failure [138]. It improved ex vivo MSCs proliferation and survival via up‐regulation of miR‐21 to suppress Fas [138, 139]. It also prolonged MSC survival in vivo and promoted MSC mobilization and homing into the infarcted myocardium partly through SDF‐1α/CXCR4 cascade [139]. In addition, atorvastatin played a beneficial impact on endothelial function [140], which facilitated the survival of MSCs and promoted the therapeutic action of MSCs in infarcted hearts via eNOS/NO and SDF-1/CXCR4 pathways [140]. Vitamin E is a well-known antioxidant for its radical scavenging activity [141]. Vitamin E- pre-activated MSCs were resistant to H_2_O_2_-induced oxidative stress along with upregulation of proliferative markers (proliferating cell nuclear antigen and Ki67) and pro-regenerative markers (TGF-β and VEGF). Moreover, implantation of MSCs with Vitamin E served to repair the damaged cartilage in a rat model of osteoarthritis [142].

In recent years, TCM or its extracts has been investigated for their beneficial effects on MSCs [143]. Salvia miltiorrhiza (SM) is a widely known herb commonly found in many prescriptions of TCM for treating various diseases, including cardiovascular disease, Alzheimer's, and ischemic stroke [144–146]. SM effectively enhanced the viability and reduced cellular damage of MSCs under hypoxic condition. The infusion of SM modified MSCs showed the infarcted areas recovery and positive behaviour changes in the rat middle cerebral artery occlusion model [147]. Curcumin, an active component of turmeric (Curcuma longa), possesses pleiotropic effects such as antioxidant and anti-inflammatory [148, 149]. It exerts cytoprotective effects against oxidative stress-induced injury in ASCs by regulating PTEN/Akt/p53 pathway and haeme oxygenase-1 expression [150, 151]. Prior curcumin treatment significantly increased VEGF secretion in MSCs, and these pre-activated MSCs resulted in more neovascularization and functional recovery than naïve ASCs in ischemic myocardium [152]. In addition, curcumin pre-activated MSCs improved their therapeutic potential in acidic burn wounds as exhibited by improved microcirculation, pronounced granulation and hastened wound closure compared with wild type MSCs [153]. There are many other extracts of TCM, such as rosmarinic acid (RA) and gigantol, which protected the MSCs against H_2_O_2_-induced apoptosis via attenuating the expression of caspase-3, caspase-9 and Bax/Bcl-2 by regulating the PI3K/Akt and ERK1/2 signalling pathways [154–156]. These results indicate that they may developed as cytoprotective agent for successful MSC transplantation.

#### Bioactive compound for enhancing the immunomodulatory, paracrine and therapeutic potential of MSCs

In fact, the immunomodulatory and paracrine properties of MSCs are closely related to their therapeutic efficacy. Recent data demonstrate that pharmacological stimulus can boost the paracrine and immunoregulation potential of MSCs. Iron chelator deferoxamine (DFX) is a hypoxia mimetic agent with antioxidant properties. DFX used to pre-activate MSCs resulted in enhanced the secretion of anti-inflammatory (IL-4, IL-5 and COX2), pro-angiogenic factors (VEGFα and Angiopoietin-1), as well as neuroprotective factors (nerve growth factor, glial cell-derived neurotrophic factor, and neurotrophin-3) in MSCs [157]. The secretome of DFX- pre-activated MSCs could effectively reprogram LPS-induced macrophage DH82 into M2 phenotype [158]. And it also showed neuroprotective potential of dorsal root ganglion (DRG) neurons under high-dose glucose-induced injury [157]. Treprostinil, a prostacyclins analogue, was used to stimulate MSCs and produce proangiogenic effects by increasing VEGF-A production [159]. Overexpression of pro-survival, angiogenic, and pro-migration related genes, including COX‐2, HIF‐1, CXCR4, CCR2, VEGF, Ang‐2 and Ang‐4, has been found in All-trans retinoic acid (ATRA) ‐treated MSCs [160]. Moreover, wounds injected with ATRA‐treated MSCs showed significantly higher levels of vascularization, collagen deposition and re-epithelialization, resulting in accelerated wound closure compared to wounds injected with untreated MSCs [160]. Resveratrol (RSV) is a plant polyphenolic compound, which can protect MSCs from inflammation and oxidative injury [161, 162], potentiating their paracrine function, preventing their ageing and so on [163, 164]. For example, RSV pre-activation enhanced the secretion of PDGF-DD in MSCs that further activated the ERK signalling pathway in renal tubular cells, promoted angiogenesis in endothelial cells, and preferably repaired cisplatin-induced renal injury [164]. Buyang huanwu decoction (BHD) is a famous formula in TCM for supplementing Qi and activating blood and has been used to treat central nervous diseases [165]. Compared with untreated BMSCs, exosomes derived from BHD- pre-activated rat BMSCs contained more angiogenetic miRNA and elevated angiogenesis in rat brain after bilateral carotid artery ligation [166].

The development of high-throughput technologies brings us a new perspective for screening bioactive compounds to target specific genes in MSC, thus regulating the expression profile of MSCs and specifically enhancing their desired biological functions. For example, tetrandrine was selected to specifically upregulate PGE2 expression in MSCs through the NF-κB/COX-2 signalling pathway. Tetrandrine- pre-activated MSCs showed a significant reduction in TNF-α secretion after co-culture with mouse macrophages (RAW264.7) and attenuation of TNF-α level in mouse inflamed ear [167].

#### Bioactive compound for reversing and repairing of disabled MSCs

MSCs from perinatal tissues will undergo replicative ageing with the large-scale amplification in vitro, and MSCs from aged donors generally present premature senescence phenotype, and MSCs from patients showed the decline of MSC biological functions. These MSCs generally present downregulated cell function in proliferation, mobility, differentiation, and immunoregulation with impaired therapeutic capability [98–100]. Therefore, reversing the functions of these MSCs, namely the rejuvenation of MSCs, is vital for MSC-based therapy. Fortunately, recent studies have shown cellular functional decline or premature senescence can be rescued [168]. In general, approaches to rescue MSCs can broadly defined as reducing the level of intracellular oxidative stress, reprograming MSCs through adjustment of epigenetic modifications, as well as usage of senolytic drugs.

##### Inhibition of excessive oxidative stress

Data show that ROS, as natural by-products produced by cell metabolism, is maintained at a low level in MSCs and is essential for the proliferation and differentiation of MSCs [169, 170]. High levels of ROS-induced under chronological ageing or pathological conditions will cause severe cytotoxicity and cell damage [168]. Several studies have reported that malfunctioned MSCs can be reversed by modulation of intracellular ROS aggregation and oxidative metabolism [168]. Antioxidants have become the natural choice, and several antioxidants used for anti-ageing studies of MSCs. N-acetylcysteine (NAC), a ROS scavenger, can significantly attenuate ROS accumulation due to overactivation of Wnt/β-catenin signalling in MSCs, thereby lessening ROS induced DNA damage and downregulating the expression of senescence-associated marker p16 (INK4A), p53 and p21 [171]. Another free radical scavenger, edaravone, rescued the functions of elderly AT-MSCs by reducing ROS level and β-gal-positive cells. Moreover, it could also protect BMSCs from the intracellular accumulation of ROS caused by hypoxia and upregulation of antioxidant enzymes in UC-MSCs [172, 173]. More importantly, pre-activation with edaravone restored the elderly AT-MSCs’ in vivo therapeutic functions as decreased necrotic area in an ischemic flap mouse model [174].

Overall, ROS can act as intracellular messengers and help perform vital biological functions, so it is essential to control the optimal concentration of ROS by adjusting the amount of antioxidants. After all, high doses of antioxidants can cause DNA damage and premature senescence [175].

##### Bioactive compound for modifying of epigenetic dysregulation

Epigenetic regulation, an important mechanism for programming, changes the cellular phenotype by alteration of gene expression rather than DNA sequence. It is characterized by heritability and reversibility, includes DNA methylation and histone modifications, and has profound influence on MSC fate [176, 177]. The epigenetic dysregulation found in MSCs after routine culture expansion appears to be unrelated to changes in global histone acetylation level, but involves histone acetylation levels at the promoters of some genes, such as TERT, Soc2, Oct4, Runx2, and ALP, which ultimately leads to cellular senescence [178]. Moreover, a general decrease in DNA methylation has reported in MSCs derived from old compared with MSCs derived from young by using a BeadChip microarray [179]. Given the reversibility of epigenetic modification, it is a potential strategy to explore epigenetic targeted therapy for reprogramming old stem cells into youthful functional stem cells.

With current techniques, methods to reprogram ageing stem cells occur in two main ways, fully reprogramming and partial reprogramming. The former is referred to the reset of the epigenetic clock for finally obtaining the induced pluripotent stem cells (iPSCs). Functional MSCs have successfully generated from iPSCs with rejuvenated gene signature and improved cell vitality, but their immunoregulatory function for suppressing T cell proliferation is incomplete [180–182]. Therefore, the therapeutic efficiency of iPSCs-derived MSCs, especially the immunomodulatory functions, needs to be thoroughly assessed. In addition, due to the low efficiency, the limited number of iPSCs, and high cost, fully reprogramming is still only a means in the laboratory, and there is still a considerable distance from the bedside. Partial reprogramming involves incomplete dedifferentiation and is considered as epigenetic rejuvenation, which can achieved by regulating DNA methylation and histone modification using bioactive compounds [183].

DNA methyltransferase (DNMT) inhibitor, 5‐azacytidine (5‐AZA), is readily incorporated into DNA and inhibits methylation patterns of specific gene regions, simultaneously activating relevant genes [184]. 5‐AZA pre-activation reversed the aged phenotype of ASCs and enhanced their proliferation, shortened population doubling time, and increased extracellular vesicle secretion via reducing ROS accumulation, ameliorating superoxide dismutase activity, and increasing BCL-2/BAX ratio [185]. Moreover, RG108 is also known as a DNA methyltransferase inhibitor. RG108-educated BMSCs showed a significantly reduction in β-galactosidase-positive cells, simultaneously with up-regulation of anti-senescence genes TERT, bFGF, VEGF, and ANG and down-regulation of senescence-related genes ATM, p21, and p53 [186].

Tetramethylpyrazine (TMP), the bioactive component extracted from the rhizome of the Chinese herbal medicine Chuanxiong, can epigenetically alleviate senescent phenotype of BMSCs by regulating EZH2 (a histone-lysine N-methyltransferase enzyme)-H3k27me3 [187]. EZH2 has found to repress transcription of both p16/p14 by increasing H3K27me3 along the Ink4A locus [188]. Moreover, previous studies have revealed that TMP also possesses the capacity to significantly delay MSC senescence by suppressing NF-κB signalling and positively regulating the proliferation, lineage commitment, and anti-apoptosis [189–191].

Increased histone acetylation, decreased DNA methylation and hydroxymethylation, and distinct changes in H3K27me3 in the genome are prevalent in senescent cells. However, reversing stem cell senescence by altering epigenetic modifications is still in infancy. It is necessary to map out the detailed epigenetic alterations of MSCs during senescence, especially the epigenetic characteristics associated with the altered behaviour in MSC biology. In addition, the universality or uniqueness of epigenetic changes in MSC ageing from different sources needs to be confirmed.

##### Usage of senolytic drugs

Senotherapeutics refers to the application of senolytic drugs to selectively deplete senescent cells or delay the onset of senescence, thereby rejuvenating tissues and reducing the occurrence of age-related pathologies [192]. Several compounds have reported to hold the perspective senolytic effects, such as navitas (ABT-263), quercetin, danazol, nicotinamide riboside, dasatinib and metformin [192, 193]. A comparative study found that ABT-263, instead of quercetin, danazol, and nicotinamide riboside, suggested possessing senolytic effects in a replicative senescence model of MSCs after long-term expansion [193]. In addition, abdominal fat-derived MSCs in pregnant women with preeclampsia present a senescent phenotype with decreased cell function and viability. Treatment of them with the anti-senescence drug dasatinib was both able to selectively promote apoptosis of senescent MSCs and dramatically improve the biological activities of MSCs, including an increase in angiogenic potential, reduction in SA-β-gal positive cells, and downregulation of IL-6, IL-8, MCP-1, and p16 [194].

It is conceivable that combinations of different drugs may achieve a more pronounced senolytic effect. As shown in the study of Zhou et al., a senolytic cocktail of dasatinib and quercetin improved osteogenic potential of aged mouse-derived BMSCs in vitro or in vivo calvarial defect model, accompanied by a decrease in SA-β-gal-staining cells, and a reduction in senescence-associated and inflammation markers including p16, p21, IL-6, MCP1, and CXCL1 [195]. Notably, several findings have shown that a cocktail of dasatinib and quercetin can reduce the burden of senescent cells and meliorate the function of vital tissues such as adipose, bone, aorta, and brain [195, 196]. However, more evidence is needed to support the effectiveness of senolytic cocktail therapies in rescuing the functions of MSCs.

Generally, these findings suggest that bioactive compounds have properties to improve disabled MSCs and repair senescent MSC. Still, their dosage, combination, and suitability for MSCs from alternative sources require in-depth exploration and verification. In addition, the exploitation of more effective bioactive compounds to rescue MSCs is also the direction that scientists need to continue their efforts. Besides, the way to use bioactive compounds is also worth careful consideration. As demonstrated in several studies, using bioactive compounds as concomitant agents for MSC transplantation also has a better therapeutic effect than stem cell transplantation alone [24, 95, 197]. Still, patients with chronic diseases are also accompanied by multiple risk factors, such as age, diabetes mellitus, and cardiovascular diseases. Hence, the possible side effects and safety of concomitant drugs for stem cell transplantation should be considered. As deficiency of MSCs can reversed, this area is a potential hotbed for increasing the longevity and biological properties of in vitro expanded MSCs, and repairing patient-derived MSC for autologous transplantation [198]. However, the senescence of MSCs is a highly complex process and a thorough understanding of the underlying mechanism of senescent will help us to find more effective ways to rejuvenate ageing MSCs.

### Pre-activation of MSCs with the disease-associated effector cells or patient's serum

“Individualized MSCs therapy” means that MSCs obtained by in vitro pre-activation possess customized functions and can specifically target the disease of the patient, thus achieving a better therapeutic outcomes. Therefore, as an enhancement strategy for MSC-based therapy, direct use of effector cells or their released active substances was proposed as pre-activating conditions, rather than educated with typical proinflammatory factors, cytokines, or bioactive compounds.

Mast cells (MCs) have a central role in immediate hypersensitivity and allergic reactions and are also the principal effector cells in the pathogenesis of atopic dermatitis (AD). Activated MCs release granules that contain a large number of bioactive substances, such as proinflammatory cytokines, protein mediators, lipid mediators, and growth factors, that can trigger allergic reactions [199]. Several studies have demonstrated that MSCs suppress MC activation and degranulation and induce MC apoptosis in a co-cultured system [200–202]. Then, pre-activated MSCs with MC granules could be a promising strategy to enhance the MSC-targeted treatment of AD. The study of Lee et al. showed that MC granule-primed UC-MSCs exhibited more immunosuppressive than non-primed cells, which are mediated by interrupting proliferation and degranulation of MCs via upregulating the COX-2/PGE2 signalling pathway [201]. In addition, in a dermatophagoides farina-induced AD model, subcutaneously infusion of MC granule-educated UC-MSCs showed a more significant decreased number of MCs and alleviated the infiltration of lymphocytes in the skin than that of naïve cells [201]. Therefore, MSCs pre-activated with effector cells or their derived active substances can accurately target the main pathogenic factors in disease development, and react and respond quickly in vivo to achieve more efficient treatment outcomes.

Alternatively, the alteration of inflammatory factors, chemokines, growth factors, cytokines, and microvesicles in blood circulation has found in a variety of diseases, including Alzheimer's disease, renal diseases, and heart disease [203–205]. They serve as indicators for disease diagnosis, treatment, and prognosis, along with inter-individual variations. Pre-activated expanded MSCs with patient-derived serum may allow MSCs to respond positively to the host microenvironment [203]. As Tang et al.'s study showed, compared with rats injected with control serum- pre-activated MSCs, rats injected with stroke serum- pre-activated MSCs showed a significantly improved behaviour with attenuated inflammatory cytokines, decreased brain lesion and apoptosis cells, and increased trophic growth factors in the cerebral I/R injury model [206]. In addition, strengthening the therapeutic effect of MSCs with disease-derived serum have also revealed in dextran sodium sulphate-induced colitis rat models. After MSCs pre-activated with serum derived from colitis rats or normal rats, the conditioned medium of both pre-activated cells was collected for treating colitis. The former shows more effectively impede the disease progress, better improvement in the clinical features, and much lower histological damage scores in colitis rats than the latter [207].

Therefore, disease-specific pre-activation may be a promising means to achieve "MSC customized clinical treatment." And accurate capturing of disease specificity is an essential prerequisite for this strategy, which requires comprehensive and in-depth exploration and analysis of the biological mechanism of diseases.

## Perspective

In addition to pre-activating MSCs by a recreation of the physiological and pathological microenvironment, there are other means for MSC pre-activation, including photostimulation, magnetoelectric stimulation, and heat shock (HSP), etc. Low levels of lasers therapy (LLLT) is beneficial for regulating the biological functions of a variety of cells [208, 209]. For example, the biological activities of ASCs stimulated by low-level laser were enhanced, manifested by increasing survival rate, augmenting secretion, and accelerating regenerative healing compared with unstimulated ASCs [210, 211]. Moreover, pulsed electromagnetic fields have recently demonstrated to play a protective effect on BMSCs through regulating the Akt/Ras signalling pathway and upregulation of survival proteins such as Bad and Bcl-xL [212]. In addition, studies have shown that HSP can induce cytoprotective proteins and increase the ability to resist a poor external environment [213, 214]. HSP pre-activation enhanced MSCs autophagy and increased their resistance to H_2_O_2_-induced apoptosis. Besides, HSP-MSCs showed enhanced homing and survival following transplantation in a hepatic I/R injury model compared with control MSCs [215]. Furthermore, intraovarian injection of HSP-MSCs rescued the damaged ovarian structure and ameliorated endocrine function [216].

Generally, the purpose of existing pre-activating approaches is to take full advantage of the functional plasticity of MSCs and assign the desired properties to MSCs in advance so that when MSC reencounters similar environment, the cell protection mechanism initiated, the response mechanism activated rapidly, and the corresponding biological response acted quickly.

Notwithstanding, the application of the pre-activated MSCs confronted with several challenges. The first is to choose reasonable and effective MSC pre-activating methods. To date, so many complementary methods have been proposed for improving the therapeutic efficacy of MSCs, and finding the best pre-activation way is an essential pursuit in our future research. Each pretreatment targets improving a specific aspect of MSCs, and the optimal combinations of diverse strategy are conceivable to maximize the therapeutic outcome of MSCs. For example, MSCs were inoculated into an injectable gel of collagen microcarriers cross linked with bFGF or TGF-β1 to promote expansion and chondrogenic differentiation [217]. Furthermore, effector cell-based or patient serum-based pre-activation of MSCs may be relatively more targeted for disease treatment, which requires amounts of comparative studies to further determine this inference. Definitely, novel enhancement strategies to generate therapeutically effective MSCs are still demanded and will undoubtedly receive constant attention.

The second is the heterogeneity of MSCs that mainly manifested in two aspects: one is that the biological characteristics of MSCs from diverse origins appear to vary in terms of differentiation, phenotype panel, and secretion profile, which means that as a novel cellular drug, MSCs from different tissues have inconsistent therapeutic effects on the same disease. As demonstrated in Liu et al.'s study, ASCs held the most pronounced effect on promoting re-epithelialization and wound closure compared with BMSCs and amnion MSCs (AMSCs). Furthermore, ASCs had the most excellent impact on enhancing the migration of dermal fibroblasts and the expression of pro-repair cytokines such as VEGF, bFGF, and TGF-β in contrast with BMSCs and AMSCs [218]. Therefore, it is necessary to gain insight into the unique biological characteristics of each tissue-derived MSCs and to determine the disease trophic properties of each MSCs in combination with different disease models. The other is the discrepancy of MSCs from different tissue in responsiveness to the same precondition, suggesting an optimal pre-activation way for various MSCs. For example, TNF-α-educated BMSCs showed significantly higher level of IL-10 than TNF-α-primed UC-MSC [219]. Further, response mechanisms of MSCs derived from different tissues vary when faced with the same pre-activating condition. For instance, co-culture of stimulated/unstimulated UC-MSCs with phytohaematoagglutinin-activated lymphocytes resulted in early activation of a negative co-stimulatory molecule CTLA4 in UC-MSCs, whereas changed IL-12 expression in co-cultured BMSCs [219]. These discrepancies could be partly determined by the functional heterogeneity between MSCs from different tissues. This may require a deeper understanding of their developmental processes and signalling regulatory networks, and a combination of high-throughput techniques and basic research can provide possible clues to determining the optimal pre-activation methods for each type of MSCs.

**Fig. 3:**
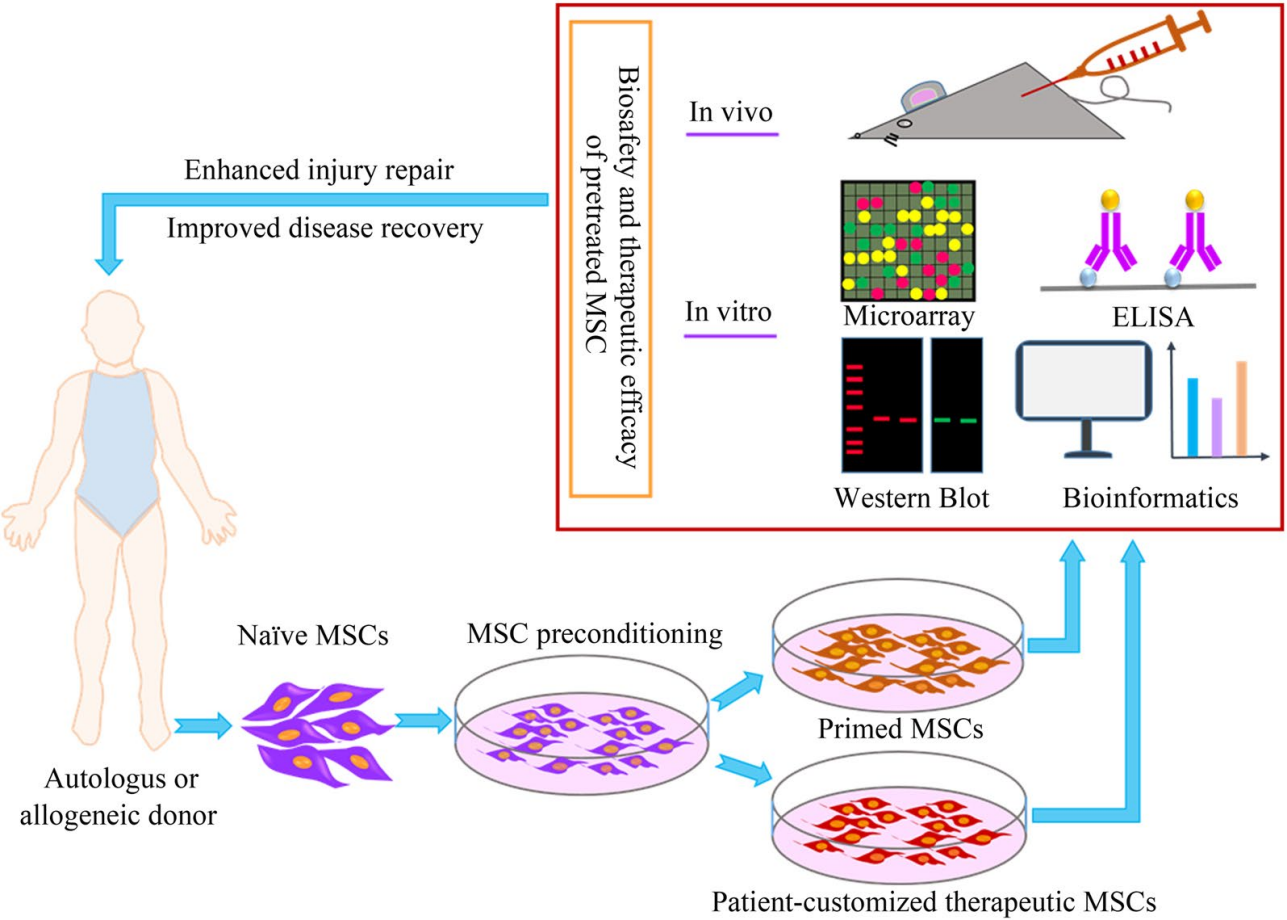
Cycle of naive MSCs to preconditioned MSCs towards clinical treatment. Preconditioned MSCs achieved favourable therapeutic efficacy, with enhanced injury repair and disease recovery

The third is to develop standard platforms for evaluating the safety and therapeutic characteristics of pre-activated MSCs (Fig. 3). Extensive research evidence confirms that MSCs mainly rely on paracrine to exert biological effects. The sum of MSCs secretion products can be regarded as the secretome, which mainly contains soluble proteins and extracellular vesicles, of which the former includes cytokines, chemokines, growth factors, and the latter can divided into exosomes and microvesicles [220, 221] (Fig. 1). Therefore, it is a troublesome problem to accurately select a component both as an indicator of preactivation and therapeutic effect of educated MSCs. However, there is increasing evidence that in vivo apoptosis of transplanted MSCs is closely associated with stem cell therapeutic effects in multiple animal models, such as GvHD [222], sepsis and acute lung injury [223, 224]. The findings of Pang et al. are further illustrated impeding MSC apoptosis by ablation of BAK/BAX reduces their immunomodulatory capacity in the model of OVA-induced asthma, suggesting that the in vivo biological mechanism of MSCs is far more complicated than we thought [225]. Therefore, inhibition and resistance to stem cell apoptosis as a commonly used evaluation index of MSC pre-activation are questionable.

Finally, the essence of MSC therapy in vivo is to deliver pro-reparative regulatory factors and extracellular vesicles, etc. Therefore, researchers have begun to use MSC-derived soluble proteins and extracellular vesicles to replace stem cells for treatment [226, 227], whose release is precisely regulated and their composition changes with the pre-activated condition [228]. This method can avoid the risk of in vivo proliferation, differentiation of MSCs, and secretion of unpredictable paracrine factors in MSCs. Recent results showed that MSCs were enucleated into "cargocytes" by density-gradient centrifugation to form a bioinspired delivery method. Cargocytes retain paracrine secretion capacities, do not proliferate, or permanently engraft in the host. Application of cargocytes not only avoids the adverse events associated with the direct use of MSCs, such as pulmonary or cerebral emboli, but also improves biodistribution and enhances homing to target tissues in vivo [229]. Therefore, derivations from the MSCs may be therapeutic vehicles to deliver curative cargos with the potential to treat diseases in a controllable and effective manner.

## Conclusion

In summary, despite pre-activated MSCs remaining problematic, they still hold considerable promise for the treatment of various refractory diseases due to their tremendous regenerative potential. To date, there is a growing consensus that pre-activated MSCs indeed exhibit better therapeutic benefits than naive MSCs in a variety of pathological conditions. In future, we should effectively use the "pre-activation" tool to maximize the therapeutic potential of MSCs on the one hand and on the other hand to modify them suitable for targeted disease, opening a new chapter for clinical application of MSCs.

## Data Availability

Not applicable.

## Abbreviations

MSC: Mesenchymal stem cell
ASCs: Adipose-derived MSCs
T2D: Type 2 diabetes
VEGF: Vascular endothelial growth factor
3D: Three-dimensional
HIF: Hypoxia-inducible factors
HPHs: HIF-1 prolyl-hydroxylases
HRE: Hypoxia-response element
HGF: Hepatocyte growth factor
BMSCs: Bone marrow-derived mesenchymal stem cells
PrPC: Cellular prion protein
bFGF: Fibroblast growth factor-basic
ANG: Angiogenin
TNF-α: Tumour necrosis factor-alpha
PGE-2: Prostaglandin E2
TGF-β1: Transforming growth factor-β1
ECM: Extracellular matrix
Hap: Hydroxyapatite
MCP-1: Monocyte chemoattractant protein-1
IL: Interleukin
SCI: Spinal cord injury
GMSCs: Gingival tissue-derived MSC
IDO: Indoleamine 2,3, dioxygenase
COX-2: Cyclooxygenase 2
LPS: Lipopolysaccharide
CXCL-12: Chemokine ligand 12
TCM: Traditional Chinese medicine
H_2_O_2_: Hydrogen peroxide
TMZ: 1-[2,3,4-Trimethoxybenzyl]piperazine
SM: Salvia miltiorrhiz
RA: Rosmarinic acid
DFX: Deferoxamine
ATRA: All-trans retinoic acid
RSV: Resveratrol
BHD: Buyang huanwu decoction
iPSCs: Induced pluripotent stem cells
5‐AZA: 5‐Azacytidine
TMP: Tetramethylpyrazine
ABT-263: Navitas
AD: Atopic dermatitis
MCs: Mast cells
HSP: Heat shock
AMSCs: Amnion MSCs
